# Expression of Carboxypeptidase A3 and Tryptase as Markers for Lymph Node Metastasis of Canine Cutaneous Mast Cell Tumors

**DOI:** 10.3389/fvets.2022.815658

**Published:** 2022-02-14

**Authors:** Tuddow Thaiwong, Juliana V. Cirillo, Jane Heller, Matti Kiupel

**Affiliations:** ^1^Veterinary Diagnostic Laboratory, College of Veterinary Medicine, Michigan State University, Lansing, MI, United States; ^2^Departamento de Patologia, Facultad de Medicina Veterinaria y Zootecnia, Universidade de São Paulo, São Paulo, Brazil; ^3^School of Animal and Veterinary Services, Faculty of Veterinary Sciences, Charles Sturt University, Wagga Wagga, NSW, Australia; ^4^Department of Pathobiology and Diagnostic Investigation, Michigan State University, East Lansing, MI, United States

**Keywords:** mast cell tumor, tryptase, carboxypeptidase A3, prognosis, canine, lymph node metastasis

## Abstract

Detection of metastatic mast cell tumors (MCTs) in lymph nodes is a critical factor for treatment, prognosis, and clinical management. Presence/absence of mast cells in the lymph nodes cannot be used as a sole parameter to determine metastasis due to the inability to differentiate neoplastic from non-neoplastic/inflammatory mast cells. While cytologic and histopathologic classifications for assessment of metastatic MCTs based on the numbers and distribution of mast cells have been developed, inconsistency between the clinical interpretation of these grading schemes and actual metastatic status occurs. The aim of this study is to identify a novel diagnostic tool to accurately predict overt metastatic mast cell tumors in lymph nodes. We investigated the possibility of using RT-qPCR to detect mRNA expression of mast cell-specific genes in lymph nodes with different stages of MCT metastatic classification. We are able to establish a highly sensitive and discriminating RT-qPCR measuring Carboxy peptidase A3 (CPA3) and tryptase mRNA expression and identify the cut-off values with high sensitivity and specificity for overt metastatic MCTs in lymph nodes. An area of future interest would be to expand our analysis of the extent to which cut-off values for these markers in correctly identifying disease status, as well as predicting clinical outcomes and survival times. This would offer valuable information regarding the practical applicability of this technique and may enable us to improve our standards of detection metastasis, including possibility of molecular analysis of cytologic specimens obtained from suspicious nodes subjected to surgical excision.

## Introduction

Mast cell tumors (MCTs) are one of the most common cutaneous tumors in dogs with a highly varied biological behavior that can range from localized disease to local invasiveness, nodal metastasis, and potentially disseminated disease (1, 2). Determining nodal metastasis of cutaneous MCTs is essential for accurate prognostication and therapeutic decision making (3–5). Cytological evaluation of regional lymph nodes in dogs with MCTs has been the most widely used method to determine nodal spread. Unfortunately, there is no standardized approach among oncologists on which node should be sampled. Moreover, sentinel nodes are not routinely examined (6). While it has been suggested that nodal examination of only enlarged regional lymph nodes should be performed, 50% of lymph nodes with metastatic MCTs will not be enlarged and would be missed by this approach (4). Because non-neoplastic mast cells will also drain to regional lymph nodes of dogs with cutaneous MCTs, detecting the simple presence of mast cells in a lymph node is an unreliable measurement of metastatic disease regardless of the sampled node. Therefore, a standardized method for predicting nodal metastasis based on cytologic examination of the node was published by Krick et al. in 2009 (7). A similar histologic classification for surgically excised lymph nodes of dogs with cutaneous MCTs that predicts the likelihood of nodal metastasis (HN0-HN3) and correlates the different classes with clinical outcome, was published by Weishaar et al. (8). However, some discrepancies have been reported between the cytologic and histologic results when determining nodal spread of cutaneous MCTs in the same lymph node (9). Such discrepancies are mainly rooted in the smaller size of cytological samples compared to extirpated lymph nodes as well as different areas of nodes being examined. More importantly, while both classification systems have established clear criteria for nodes with overt metastasis as well as nodes with no evidence of metastatic MCT spread, both systems classify a significant number of cases as suspect metastases with varying degrees of certainty. In fact, the terms “pre-metastasis” and “early metastasis” for the HN1 and HN2 categories are inaccurate, as neither category has been confirmed to progress to overt metastasis. Instead, both categories represent different degrees of suspicion of metastatic disease with different associated outcomes. Lastly, both classification systems are hampered by a number of technical limitations as neither the number of samples taken from each node for cytologic examination nor a standardized trimming method for excised nodes have been established (10). Thus, a more standardized approach that can identify nodal MCT metastasis with a higher degree of certainty is urgently needed.

The identification of biomarkers for neoplastic cells may yield novel tools to predict nodal metastases more accurately. In humans, genes that are most consistently overexpressed in patients with mastocytosis include tryptase and carboxypeptidase A (11). Tryptase is primarily restricted to mast cells and can constitute as much as 23% of mast cell proteins (12). Human mast cells are so abundantly endowed with tryptase that tryptase has emerged as perhaps the most sensitive and specific means of detecting mast cells in tissues and biopsies (13). Detection of tryptase in blood is widely used as a biomarker for human mastocytosis, anaphylaxis risk, and mast cell activation (14–19). In dogs, detection of tryptase expression with immunohistochemistry has shown excellent specificity and sensitivity for mast cells; however, the immunohistochemical labeling patterns were not significantly associated with prognosis (20). Furthermore, it has been demonstrated that in dogs tryptase concentrations are directly related to mast cell density, which suggests tryptase as a potential marker for MCTs (21, 22). Another enzyme that is highly expressed in mast cells is carboxypeptidase A3 (CPA3) (23), originally named mast cell carboxypeptidase A, which is also expressed in basophils and some T-cell progenitor and thymic T cells (24). CPA3 is a Zn-containing metalloprotease stored in enzymatically active form. Upon mast cell activation and degranulation, CPA3 together with chymases and tryptases interacts with heparin proteoglycans (25). The CPA3 transcript is highly upregulated and readily detected in human mastocytosis as well as in mast cells infiltrating various human neoplasms (26), making it a potentially useful biomarker for detecting neoplastic mast cells. There are no specific data for its expression in canine MCTs. The goal of this study was to measure expression levels of tryptase and CPA3 as potential markers for lymph node metastasis of canine cutaneous MCTs.

## Materials and Methods

### Case Selection

Formalin fixed, paraffin-embedded tributary lymph nodes from 78 dogs with a previously confirmed diagnosis of cutaneous MCTs were retrieved from the archives of the Universidade de São Paulo and the Michigan State University Veterinary Diagnostic Laboratory. The diagnosis of cutaneous MCTs was confirmed for all cases through review of hematoxylin and eosin (HE) stained slides of the associated skin biopsy by two board certified pathologists. The C2 canine MCT cell lines that harbor an internal tandem duplication in exon 11 was used as a positive control. Thirteen lymph nodes from dogs with no evidence of MCTs or any other neoplastic disease (5 dogs with allergic dermatitis and 8 dogs with hyperplastic lymph nodes) were included as negative controls.

### Histological Examination and Classification of Nodal Metastasis

Serial sections of all lymph node specimens at a thickness of 5 microns were routinely stained with hematoxylin and eosin for microscopic evaluation as well as with Toluidine blue to determine the number and distribution of mast cells within lymph nodes. Lymph nodes were classified according to the HN0-HN3 classification system by Weishaar et al. (8). Briefly, HN0 (no metastasis) is characterized by none to <3 scattered individualized mast cells in sinuses per 400X field. HN1 (pre-metastasis) is characterized by >3 individualized mast cells in sinuses and/or nodal parenchyma in a minimum of four 400X fields. For HN2 (early metastasis), the lymph node has to contain clusters of more than 3 associated mast cells in sinuses and/or parenchyma, and, in HN3 (overt metastasis), there is disruption or effacement of normal nodal architecture by discrete foci, nodules, sheets, or overt masses of mast cells.

### RNA Extraction and RT-qPCR

Total RNA was isolated from formalin fixed, paraffin embedded lymph nodes using the RNeasy FFPE Kit (QIAGEN, catalog#73504) according to the manufacturer's protocol. Following deparaffinization with CitroSolv, lymph node tissue was incubated in proteinase K containing lysis buffer. The RNeasy MinElute spin column binds final total RNA, which was then eluted in 40 mL of water. RNA was quantifed using Qubit (ThermoFisher Scientific, catalog#Q32852). One microgram of total RNA was treated with TURBO DNA-free™ (ThermoFisher Scientific, catalog#AM1907) to remove contaminating DNA. First-strand cDNA synthesis was performed using SuperScript III Reverse Transcriptase (ThermoFisher Scientific, catalog#18080044) with random primers (Promega, catalog#C1181). The cDNA was then column-purified by using QIAquick PCR Purification Kit (QIAGEN, catalog# 28104), and eluted with distilled nuclease-free water at 10 ng/μl.

For determination of specific gene expression, each primer was designed with Primer3 software (27, 28). The primers for beta 2 microgloculin (β2MG), Chymase, CPA3 (Ensembl:), Fc fragment of IgG receptor Ia (FCGR1A), KIT, Prostaglandin D2 Synthase (PTGDS), and tryptase were listed in Table 1.

**Table 1:** Primer sequence used in this study.

**Gene**	**Source**	**Primer**	**Sequence**	**Size(bp)**
β2MG	ENSCAFG00000013633	Forward	5′- CCT TGC TCC TCA TCC TCC TC-3′	129
		Reverse	5- ACC CTG ACA CGT AGC AGT TC-3′	
Chymase	ENSCAFT00000019746	Forward	5′-TCT GCA AGA GGT GAA GCT GA-3′	192
		Reverse	5′-TTT GCA TCA TTC TGC CCA TA-3′	
CPA3	ENSCAFT00000013074	Forward	5′-AAA CTC CTG GAC CGA ATG AAT-3′	148
		Reverse	5′-AGT TCC TGT TGA GGT CAG TGC-3′	
FCGR1A	ENSCAFG00000011504	Forward	5′-TGG TGA ATA CAG GTG CCA GA-3′	143
		Reverse	5′-TCC ATC CAT GAC ACC TCA AA-3′	
KIT	ENSCAFG00000002065	Forward	5′-GGA AGA TGA TGA GTT GGC TCT-3′	143
		Reverse	5′-TTC GAC CAT GAG TAA GGA GGA-3′	
PTGDS	ENSCAFG00000019533	Forward	5′-GAC CAG TGT GAG ACT CGA ACC-3′	128
		Reverse	5′-GCG TAC TCC TCG TAG TTG GTG-3′	
Tryptase	ENSCAFG00000031939	Forward	5′-CGT CGT GTG TCC TGA AGA AAT-3′	121
		Reverse	5′-CCC ATT CTC GGG TGT GTA GTA-3′	

Ten nanograms of cDNA were used as the template in the reaction mixture for quantitative real-time PCR, which was performed on a CFX96 Polymerase Chain Reaction Detection System (BioRad) using SYBR Green (ThermoFisher Scientific, catalog# 4309155) according to the manufacturer's recommendations. The protocol was as follows: initial denaturation at 95°C for 30 s, followed by 40 cycles of denaturation at 95°C for 5 s, annealing at the temperature suitable for each gene marker for 10 or 20 s, and extension at 72°C for 10 s. The baseline was set automatically, and the threshold Cq was defined as the number of cycles in which the fluorescence exceeded the automatically set threshold. RNA from lymph node with HN0 was chosen as a calibrator, and the ratio of each target gene to the beta-2 microglobulin (B2M) expression for each tumor sample was normalized by the same ratio for the normal tissue sample using the delta-delta Cq (ΔΔCq) method. Each sample was assayed in triplicate. A control and a reference were included in every run.

### Statistical Analysis

The diagnostic accuracy of each individual mRNA marker was evaluated by sensitivity (the probability that the mRNA marker is positive given that histological examination reveals the presence of metastasis) and specificity (the probability that the mRNA marker is negative given that the lymph node is histologically uninvolved). Difference between the expression levels of CPA3 and tryptase in lymph nodes from each nodal status were analyzed by Student's *t*-test. To set cutoff values for relative RNA levels, receiver operating characteristic (ROC) curve analysis was performed by plotting the true-positive fraction (sensitivity) and false-positive fraction (specificity) pairs with area under the curve (AUC) values for lymph nodes grouped according to the histologic HN0-HN3 classification by Weishaar et al. (8). Differences between groups were compared. A *P*-value < 0.05 was considered statistically significant.

## Results

According to the HN0-HN3 classification by Weishaar et al. (8), 17 lymph nodes were classified as HN0, 20 nodes as HN1, 20 nodes as HN2, and 21 nodes as HN3.

### Overexpression of CPA3 and Tryptase in Mast Cell Tumors

Preliminary tests analyzing the mRNA expression levels of six genes were performed first. We found that expression levels of four genes, namely Chymase, FCGR1A, KIT, and PGD2 synthase, demonstrated no significant differences between normal lymph nodes and lymph nodes (HN0) when compared to lymph nodes with overt metastatic disease (HN3). These genes were therefore excluded from further investigation.

Expression levels (relative cDNA levels) of CPA3 and tryptase were presented as ratios of each marker to the internal reference gene (β2MG), which provided a normalization factor for the amount of cDNA. These two candidate markers were selected based on their remarkable overexpression in the C2 canine MCT line and absence of expression in lymph nodes with no evidence of metastatic disease.

### Association Between HN Grading and mRNA Expression of CPA3 and Tryptase

**Figure 1:**
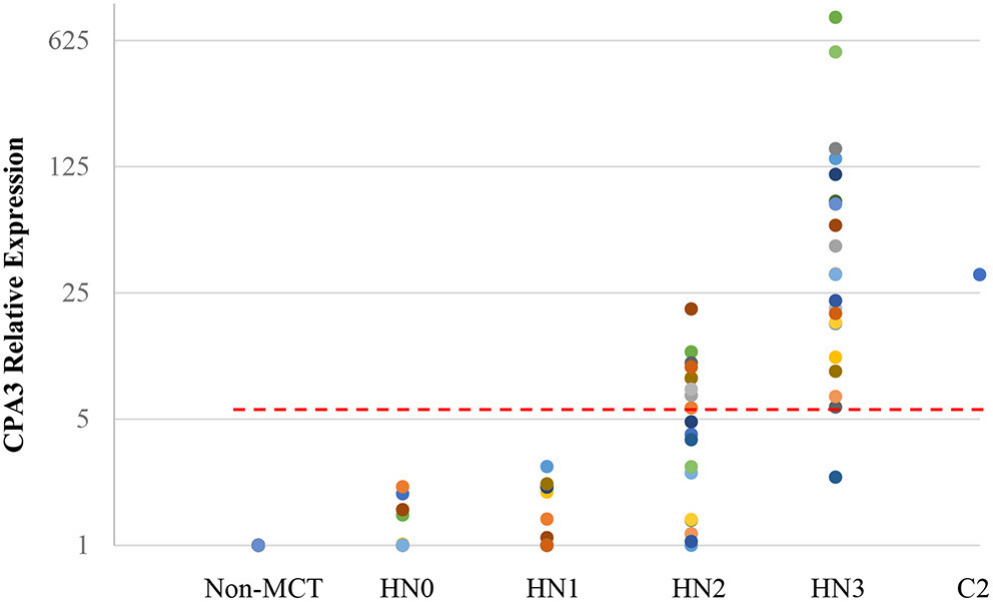
Relative mRNA expression of *CPA3* in each HN group determined by quantitative RT-PCR. Symbols showed mRNA levels in 17 LNs with HN0, 20 LNs with HN1, 20 LNs with HN2, and 21 LNs with HN3 compared with mixed HN0 LNs used as a calibrator. Red lines showed cut-off value at 5.79.

**Figure 2:**
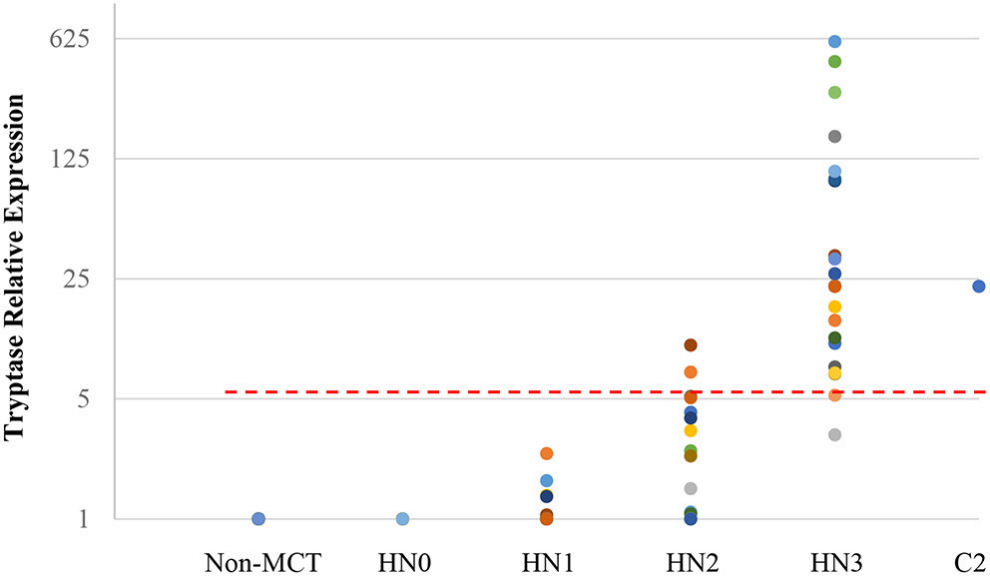
Relative mRNA expression of Tryptase in each HN group determined by quantitative RT-PCR. Symbols showed mRNA levels in 17 LNs with HN0, 20 LNs with HN1, 20 LNs with HN2, and 21 LNs with HN3 compared with mixed HN0 LNs used as a calibrator. Red lines showed cut-off value at 5.20.

Quantitative RT-PCR (RT-qPCR) was performed to quantify CPA3, tryptase, and B2MG in 17 nodes with no evidence of metastasis (HN0), 20 nodes with pre-metastasis (HN1), 20 nodes with early-metastasis (HN2), 21 nodes with overt-metastasis (HN3), and 13 control nodes with non-tumor dogs. The results are shown in Figures 1, 2. For CPA3, the expression levels were positively associated with a diagnosis of HN2 or HN3. CPA3 mRNA expression was significantly different in lymph nodes diagnosed as HN0 compared to nodes diagnosed as HN2 (*P* < 0.001) or HN3 (*P* = 0.040), respectively, as well as lymph nodes diagnosed with HN1 vs. nodes diagnosed with HN2 (*P* < 0.001) or HN3 (*P* = 0.026). The expression levels of tryptase were also significantly up-regulated in lymph nodes diagnosed with HN2 (*P* = 0.007) or HN3 (*P* = 0.019) compared to lymph nodes diagnosed with HN0. Similar differences were observed for tryptase expression levels when comparing lymph nodes diagnosed as HN1 vs. HN2 (*P* = 0.007) or HN3 (*P* = 0.011). Moreover, the mRNA expression levels of both, CPA3 and tryptase were significantly higher in HN3 lymph nodes than in HN2 lymph nodes (*P* = 0.033 and 0.013, respectively). On the other hand, no difference in the expression levels of CPA3 or tryptase were found between HN0 and HN1 lymph nodes (*P* = 0.354 and 0.0941, respectively) and between HN0 and non-MCT lymph nodes (*P* = 0.081 and 0.275, respectively).

### Diagnostic Cut-Offs for mRNA Expression Levels

ROC analysis was performed using relative expressions of lymph nodes with metastatic mast cell tumors according to the Weishaar's classification to set the best cut-off values in RT-qPCR. The best cut-off values of CPA3 and tryptase for HN3 determined by the ROC analysis were set at 5.79 (Figure 1) with 95% sensitivity and 86% specificity rates, and 5.20 (Figure 2) with 95% sensitivity and 96% specificity rates, respectively. When combining the results for CPA3 and tryptase multiplicatively, the sensitivity and specificity reached 100 and 93%, respectively, using a cut off value of 32.15, for the diagnosis of overt nodal metastasis. Applying the cut-off level at 95% sensitivity of CPA3 to all examined lymph node samples, we identified 12/20 of HN2 and 20/21 of HN3 lymph nodes as metastatic disease, whereas 2/20 of HN2 and 20/21 of HN3 were identified as metastatic disease when the cut-off value of tryptase at 95% sensitivity was applied. None of the HN0 and HN1 lymph nodes had RNA expression levels above the cut-off values of either CPA3 or tryptase.

## Discussion

The goal of this study was to develop a quantitative method to more accurately detect nodal metastasis of canine cutaneous MCTs and to reduce the large number of nodes classified with uncertainty of metastasis (HN1 and HN2) according to the current system by Weishaar et al. (8). Therefore, for the first time, we established a molecular method to detect CPA3 and TPS expressions using RT-qPCR in lymph nodes from dogs with cutaneous MCTs.

Based on the data presented here, we were not only able to consistently identify lymph nodes with histologically overt metastatic disease (HN3 nodes) through molecular testing, but also to more accurately determining the likelihood of metastatic disease in lymph nodes that had been classified based on morphologic criteria as having suspect metastatic disease of varying risks (HN1 and HN2 nodes). Less than 5% (1/21) of HN3 nodes had an mRNA expression level of CPA3 and tryptase below the cut-off values, and metastatic disease would have been undetected by either molecular test. At 95% sensitivity, analysis of expression of CPA3 and tryptase RNA levels in HN2 nodes identified 8/20 (40%) and 2/20 (10%) of these nodes, respectively, as harboring metastatic disease. Finally, we analyzed the results of the combined analysis of CPA3 and tryptase expression levels. When these levels were multiplied and applied against a cut off value of 32.15, they gave 100% sensitivity and 93% specificity. Such a high level of sensitivity means that if the multiplied values do not exceed 32.15, then metastatic disease can be ruled out very effectively.

The accurate identification of lymph nodes with mast cell tumor metastases is negatively impacted by our inability to differentiate neoplastic from non-neoplastic/inflammatory mast cells based on morphologic criteria alone. This has resulted in classification systems that identify large numbers of lymph nodes with varying degrees of potential risk for metastatic disease. In our study more than 50% of included cases were classified as HN1 or HN2, thereby presenting a high percentage of cases with diagnostic uncertainty. By analyzing RNA expression levels of CPA3 and tryptase, we were able to increase the diagnostic sensitivity and specificity to more than 95% for these cases.

Furthermore, both cytologic and histologic examination to detect mast cell granules in drained lymph nodes by H&E or special staining, such as Giemsa or Toluidine blue staining, can only evaluate a small portion of the affected lymph node. Routinely, histologic sections are cut at 5 μm thickness thereby covering <0.5% of the volume of most lymph nodes. A mast cell has a diameter of ~10 μm, and identification of individual mast cells within a 1 cm lymph node would require 1,000 serial sections, something that is simply impossible in practice. In contrast, quantitative real-time RT-PCR allows for quantification and high through-put analysis of whole lymph nodes and has become the technology of choice for the measurement of gene expression levels. In humans, RT-PCR has been used as a more sensitive method for detection of tumor cells in lymph nodes in many tumor types (29–33). When diluting RNA extracted from the C2 canine MCT cell lines within RNA extracted from a normal lymph node, we were able to detect levels of CPA3 and tryptase above the cut-off values at a dilution of 1:100,000 (10 fg), thus highlighting the high diagnostic sensitivity.

Although quantitative real-time RT-PCR to analyze gene expression profiles has certain limitations, as most tumor markers can also be expressed in normal lymph nodes, this problem has been overcome in human medicine by analyzing larger panels of markers and by evaluating the expression of multiple such markers simultaneously (32). In our study we were able to improve the accuracy of the test to a sensitivity and specificity above 95% by analyzing CPA3 and tryptase in combination.

While the proposed classification system for nodal metastasis by Krick and Weishaar allowed a more standardized reporting of suspected metastatic disease, the associated survival times are of limited clinical usefulness. Reported median survival times vary between dogs with HN3/HN2 and HN1/HN0 lymph nodes from 804 days in dogs with HN3/2 lymph nodes to 1,824 days in dogs with HN1/0 lymph nodes. By establishing more specific cut-off values that predict true metastatic disease using RT-qPCR for CPA3 and tryptase, a better clinical correlation would be expected. Future studies to test this hypothesis and to apply the proposed method in a clinical setting with associated outcome data are essential to establish this novel test in routine practice.

## Data Availability Statement

The original contributions presented in the study are included in the article/supplementary material, further inquiries can be directed to the corresponding author.

## Author Contributions

TT contributed materials, designed study, performed experiment, analyzed data, and wrote manuscript. JC contributed materials and revised manuscript. JH analyzed data, provided statistical analysis, and revised manuscript. MK contributed materials, designed study, analyzed data, and wrote manuscript. All authors contributed to the article and approved the submitted version.

## Conflict of Interest

The authors declare that the research was conducted in the absence of any commercial or financial relationships that could be construed as a potential conflict of interest.

## Publisher's Note

All claims expressed in this article are solely those of the authors and do not necessarily represent those of their affiliated organizations, or those of the publisher, the editors and the reviewers. Any product that may be evaluated in this article, or claim that may be made by its manufacturer, is not guaranteed or endorsed by the publisher.
